# Antinocieptive and anti-inflammatory effects of Toddalia asiatica (L) Lam. (Rutaceae) root extract in Swiss albino mice

**DOI:** 10.11604/pamj.2013.14.133.2130

**Published:** 2013-04-06

**Authors:** Hellen Nyambura Kariuki, Titus Ikusya Kanui, Abiy Yenesew, Nilesh Patel, Paul Mungai Mbugua

**Affiliations:** 1 University of Nairobi, P. O. Box 30197 (00100) Nairobi, Kenya; 2 School of Agricultural & Veterinary Sciences, South Eastern University College P.O. BOX 170-90200 Kitui, Kenya; 3 The Presbyterian University of East Africa, P.O Box 387 (00902) Kikuyu, Kenya

**Keywords:** Toddalia asiatica, root extract, formalin test, carrageenin test, Antinociceptive, Anti-inflammatory, mice

## Abstract

**Introduction:**

*Toddalia asiatica* is a commonly used medicinal plant in East Africa for the management of pain and inflammatory conditions. The present study investigated the antinociceptive and the anti-inflammatory effects of *T. asiatica* in Swiss albino mice.

**Methods:**

The antinociceptive and the anti-inflammatory effects of *T. asiatica* were investigated using formalin-induced pain test and the carrageenin-induced oedema paw. The extract solvent (vehicle), aspirin and indomethacin were employed as negative and positive controls respectively. Eight mice were used in each experiment.

**Results:**

In the early phase of the formalin test, the 100mg/kg dose showed no significant antinociceptive activity while the 200mg/kg showed significant (p < 0.01) antinociceptive activity. The 100 mg/kg dose showed highly significant antinociceptive activity (p < 0.001) in the late phase of the formalin test while the 200mg/kg dose showed no significant antinociceptive activity. A reduction in carragenin induced acute inflammation paw oedema was significant (p < 0.01) following administration of 100mg/kg dose but not with the 200mg/kg dose.

**Conclusion:**

The present study therefore lends support to the anecdotal evidence for use of *T. asiatica* in the management of painful and inflammatory conditions.

## Introduction

Pain as a sensory modality, represents the symptom for the diagnosis of several diseases conditions and has a protective function. Pain is widely accepted as one of the most important determinants of quality of life because of its widespread adverse effects, including diminishing mental health and wellbeing and impairing the individual's ability to perform daily activities [1]. Chronic pain impacts upon a large proportion of the adult population, including the working age population, and is strongly associated with markers of social disadvantage [1]. For thousands of years, medicine and natural products have been closely linked prominently through the use of traditional medicines. Clinical, pharmacological, and chemical research on these traditional medicines, which are derived predominantly from plants, are the basis of many therapeutic agents. Medicinal plants contain a diversity of biologically active compounds that belong to different natural product chemical classes and man throughout history has used many different forms for relief of pain. Developing treatments for pain relief has been the motivating factor behind many studies carried out in response to the demand for powerful analgesics and anti-inflammatory that exhibits their pharmacological response through new mechanisms of action and with fewer side effects [2].

*Toddalia asiatica* (L) Lam. (Rutaceae), also known as Wild Orange tree, is a green leafy climber growing in the evergreen forests and is vastly distributed in the tropical regions of Africa, Asia and Madagascar. It contains coumarins, quinoline and benzophenanthridine alkaloids. The alkaloids of the crude extract have been shown to have anti-inflammatory effects in rats using the carragennan test [3] and to inhibit the auricle swelling caused by xylol and joint swelling caused by agar in rats [4] *T. asciatica* has been shown to have anti-malarial and anti-leukimatic properties [5, 6]. The central and peripheral antinociceptive effects of *T. asciatica* have been demonstrated using mice [7]. Roots as well the leaves are used in parts of East Africa for the management of neuropathic and inflammatory pain [5, 8]. Roots have been shown to be potent in antinociception than leaves [7]. Most of the folkloric uses of the genus *Toddalia asiatica* evolve around pain, inflammation and microbial infections.

This study was undertaken to assess the analgesic actions of *Toddalia asiatica* root extract using the formalin test and carragenin induced paw oedema.

## Methods

### Plant acquisition and preparation of the extracts

Plant samples were collected from the Ngong forest area in Nairobi in February 2008 and were botanically authenticated by the University of Nairobi Herbarium and a voucher specimen deposited with the herbarium. The air-dried and powdered roots of *Toddalia asiatica* (250 g) were extracted using CH_2_Cl_2_/MeOH (1:1) for 1hour on day one and 24 hours for two sessions on the following two days at room temperature. The three extracts were combined and the removal of the solvents from the extract was done by rotatory evaporation process yielding 40 g of brown residue which was dissolved in 5% dimethylsulfoxide (DMSO) and 95% normal saline to achieve the desired working concentrations. The vehicle constituted of 5% DMSO and 95% normal saline.

### Animals

Adult Swiss albino mice of both sexes weighing 20-26 g were used. The animals were housed in cages with food and water *ad libitum*. The animal house was maintained at a temperature (20 -22deg;C) and with controlled lighting (12 h light/dark cycles). Habituation to the equipment was done 24 hours, before the commencement of the experiments and the “Principle of Laboratory Animal Care” (NIH publication No. 85-23) guidelines and procedures were followed in this study [9]. All the tests were carried out during the daytime in a quiet laboratory setting with ambient illumination and temperature similar to those of the animal house. Animals were allowed to acclimatize to the test laboratory setting for 1 hour before the experiments began. All effort was made to minimize animal suffering and to reduce the number of animals used.

### Standard drug

The reference drugs used were: (Disprin^®^ {acetylsalicyclic acid - ASA})-(Reckitt Benckiser) Batch number 801050, Indomethacin (Sigma, East Africa) Batch number 041206 (Formalin (Sigma), Batch number 040812 and Carrageenin (Sigma, East Africa) Batch number BGK 1080.

### Administration

Extracts, standard drugs and vehicle (5% DMSO and 95% normal saline) were injected 40µl intraperitoneally (i. p.) using a 17 gauge needle. Three dose levels of the extract, (50, 100, and 200 mg/kg), were selected from the pilot study.

### Bioassays

**Sensorimotor test:** To evaluate possible nonspecific muscle relaxant or sedative effects of the extract of *Toddalia asiatica*, animals were tested on an apparatus that consisted of 3 rods, diameter 2.5 cm, with the height of 20, 32, and 64 cm. Animals were placed on top of each rod for 20 seconds to test their sensorimotor function. The animals were habituated 24 hours prior to testing. Animals were treated with root bark extract of *Toddalia asiatica* (50,100 and 200 mg/kg, 1 hour prior to the test). Control animals received the same volume of vehicle (5% DMSO in 95% (0.9%NaCl) solution) i. p. 1 hour before the test. The cut-off time used was 20seconds.

**Formalin- induced licking:** Swiss albino mice (20-26 g) were treated with indomethacin (50mg/kg), aspirin (100mg/kg), vehicle and *T. asiatica* extract (50, 100, and 200mg/kg, i. p.) 1 hour before formalin injection. The procedure was similar to that described previously [10, 11]. Using a microliter syringe and a 26 gauge needle, 50µl of 5% formalin in 0.9% Normal saline was injected subcutaneously into the dorsal side of the right hind paw of each animal. The animal was returned to the observation chamber immediately after injection and the observation period started. The amount of time (in seconds) the animal spends licking and/or lifting the injected paw was recorded using a stop watch in 5 minutes blocks for a period of thirty minutes. The animals’ behavior was also recorded in terms of whether it was active, quiet or asleep during the period of the experiment. The amount of time spent licking the injected paw was scored.

**Carrageenan-induced paw oedema:** This test was carried out as described by Boughton-Smith et al. [12]. Adult Swiss albino mice of either sex weighing 20 - 26 g were treated with indomethacin (50mg/kg), aspirin (100mg/kg) vehicle and *Toddalia asiatica* root extract (50, 100, and 200mg/kg, i. p.) 1 hour before injecting carrageenin (0.1 ml of 1% suspension in 0.9% saline) in the subplantar region of the right hind paw. Two hours after injection the animals were euthanised by placing them in a glass jar with cotton wool soaked in chloroform. The paws were immediately amputated above the ankle (at the point where the fur starts to thicken) and weighed (mg) on an analytical balance. Oedema was determined by the increase in weight of the right hindpaw compared to the left hind paw.

### Statistical analysis

Data obtained for each set of experiments/tests were pooled and analysis was done using one-way ANOVA followed by Shaffes *post-hoc* test. The differences in the test- versus control-values were considered to be statistically significant at P < 0.05. Data is expressed as mean plusmn; S.E.M. The dose was the independent variable.

## Results

### Sensorimotor test

The extract of *T. asiatica* (50, 100 and 200 mg/kg, i. p.), given 1 h prior to sensorimotor testing, did not affect the motor performance of animals when compared with the control group response. All the animals were able to hold on to the three rods.

### Formalin test

Injection of the root bark extract (50, 100 and 200 mg/Kg i. p.) given 1 hour prior to the formalin test showed a significant antinociceptive effect (p < 0.01) in the early phase at 200 mg/kg dose and a highly significant antinociceptive (p < 0.001) effect in the late phase at 100 mg/kg (Table 1). The 200 mg/kg dose showed no effect in the late phase of formalin test (Table 1).

**Table 1:** Effects of *T. asiatica* root bark in the early phase (0-5mins) and the late phase (15-30 mins.) of the formalin test using mice

		Time spent in pain behavior (secs.)	
Treatment	Dose (mg / kg)	Early phase (0-5min)	Late phase (15-30min)
**Vehicle**	0	221.84 ± 10.56	190.90 ± 7.47
***T. asiatica*** **root bark extract**	50	207.54 ± 9.21	202.10 ± 21.29
	100	212.06 ± 7.64	110.58 ± 8.45***
	200	181.68 ± 4.21**	183.54 ± 11.38
**Indomethacin**	50	221.37 ± 6.71	25.33 ± 4.05***
**Acetyl salicylic acid**	100	166.70 ±7.37***	153.31 ± 10.96

Each group represents the mean ±SEM of 8 animals.

** p < 0.01

*** p < 0.001when compared with the control value subsequent to ANOVA

### Carrageenin-induced paw oedema test

Administration of the root bark extract (50, 100 and 200 mg/Kg i. p.) given 1 hour prior to the test caused a significant anti-inflammatory effect (p < 0.01) at dose 100 mg/kg (Table 2).The 50 and the 100mg/kg dose caused no significant effect. The percentage oedema inhibition of the root bark extract (100 mg/kg) was 37.04% compared to the reference drug indomethacin (50 mg/kg) at 40.74% compared to the control values (Table 2).

**Table 2:** Effects of *T. asiatica* root bark extract (50, 100 and 200 mg / kg i. p.) indomethacin (50 mg / kg), ASA (100mg/kg) and vehicle on paw oedema in the carrageenin test

Treatment	Dose (mg/kg)	Carrageenin (mice)	Oedema inhibition (%)
**Vehicle**	0	3.375 ± 0.26	-
***T. asiatica*** **root bark extract**	50	3.125 ± 0.35	3.70
	100	2.125 ± .013**	37.04
	200	2.750± 0.16	18.52
**Indomethacin**	50	1.875 ± 0.3***	40.74
**Acetyl salicylic acid**	100	3.125 ± 0.23	7.40

Each group represents the mean ± SEM of 8 animals.

** p < 0.01

*** p < 0.001when compared with the control value (ANOVA)

## Discussion

Inflammatory processes involve the release of several mediators and substances that regulate adhesion of molecules and the processes of cell migration, activation and degranulation [13]. Various models of nociception and inflammatory reactions have been used in animal models to demonstrate various analgesic and anti-inflammatory effects of phytomedicines used in the traditional healthcare system for the management of pain and inflammation. The results of the present study indicate that the root bark extracts of *T. asiatica* possess antinociceptive activity in chemical, thermal, and inflammatory models of pain. Furthermore, the plant extracts showed anti-inflammatory activities in carrageenin-induced paw oedema. From the present study, it is evident that in both the analgesic and anti-inflammatory test models used, the effects of the extract showed dose-dependent and significant effects. The effects were comparable to those of the reference drugs used ASA, morphine and indomethacin respectively. The observed analgesic and anti-inflammatory effects of *T. asiatica* root bark extract could be due to the presence of biologically active chemical compounds in the extracts.

Formalin test is widely used in the assay of antinociceptive activity [14]. Centrally acting analgesics have been known to have an effect on both the early and the late phase of the test whereas peripherally acting analgesics have an effect on the early phase only [15, 16]. This is as a result of the release of various neurotransmitters including glutamate and aspartate in the dorsal horn after formalin injection [17]. Therefore the early phase of the formalin test represents the transmission of nociceptive impulses while the second or late phase represents the events of central sensitization and wind-up phenomena [14].

In this study, the extracts of *Toddalia asistica* showed significant antinociceptive activity in the early and the late phases of the formalin test. The (200 mg/kg) caused significant antinociceptive effects in the early phase of formalin test while the (100 mg/kg) extract caused a highly significant antinociceptive effect in the late phase. This was comparable to that of the reference drugs indomethacin (50 mg/kg) and ASA (100mg/kg). The 200mg/kg dose of the root bark extract showed no significant antinociceptive effects in the late phase of formalin test. The results obtained from the formalin test suggest that *T. asiatica* root bark extract has both peripheral and central sites of action. The lower dose (100 mg/kg) was effective in the late phase and the higher dose (200 mg/kg) being effective in the early phase. There was no statistically significant difference between the 200mg/kg of the extract and the control group in the formalin test late phase. The data therefore supports the traditional/folkloric use of the plants as well as validates their use in the management pain and inflammatory conditions [18, 19].

The antinflammatory effects of *T. asiatica* root bark extract in the late phase of formalin test are further supported by the results obtained in the carrageenin induced paw oedema test which exhibited a low -dose significant anti-inflammatory effects. The inhibitory effects of the extract on acute oedema induced by carrageenin in the right hind limb paws of mice may be due, at least in part, to enzyme inhibition, chemical mediators released during inflammation and/or a reduction of white blood cell (WBC) movement into the site of injury [20].

## Conclusion

The root bark extract of *T. asiatica* showed antinociceptive and antinflammaoty activities in the animal models of pain. This supports the anecdotal use of *T. asiatica* in the management of pain and inflammation.
